# ECMO Support in Refractory Cardiogenic Shock: Risk Factors for Mortality

**DOI:** 10.3390/jcm11226821

**Published:** 2022-11-18

**Authors:** Sasa Rajsic, Robert Breitkopf, Zoran Bukumiric, Benedikt Treml

**Affiliations:** 1 Department of Anesthesia and Intensive Care Medicine, Medical University Innsbruck, 6020 Innsbruck, Austria; 2 Institute of Medical Statistics and Informatics, Faculty of Medicine, University of Belgrade, 11000 Belgrade, Serbia

**Keywords:** extracorporeal life support, ECMO, cardiogenic shock, adverse events, mortality, risk factors

## Abstract

Background: Veno-arterial extracorporeal membrane oxygenation (va-ECMO) is a specialized temporary support for patients with refractory cardiogenic shock. The true value of this potentially lifesaving modality is still a subject of debate. Therefore, we aimed to investigate the overall in-hospital mortality and identify potential risk factors for mortality. Methods: We retrospectively analyzed the data of 453 patients supported with va-ECMO over a period of 14 years who were admitted to intensive care units of a tertiary university center in Austria. Results: We observed in-hospital mortality of 40% for patients with refractory cardiogenic shock. Hemorrhage, ECMO initiation on weekends, higher SAPS III score, and sepsis were identified as significant risk factors for mortality. Hemorrhage was the most common adverse event (46%), with major bleeding events dominating in deceased patients. Thromboembolic events occurred in 25% of patients, followed by sepsis (18%). Conclusions: Although the rates of complications are substantial, a well-selected proportion of patients with refractory cardiogenic shock can be rescued from probable death. The reported risk factors could be used to increase the awareness of clinicians towards the development of new therapeutic concepts that may reduce their incidence.

## 1. Introduction

Veno-arterial extracorporeal membrane oxygenation (va-ECMO) is used as a last rescue in refractory cardiogenic shock with failing conventional therapy [1]. It may be used in different clinical presentations, including extracorporeal-assisted rewarming, resuscitation of traumatized patients, and bridging to heart or lung transplants. The initiation of ECMO in selected patients is recommended by the Extracorporeal Life Support Organization (ELSO) when the risk of mortality reaches 80% [2]. However, the true advantage of this potentially lifesaving support remains a subject of debate, and severe cardiogenic shock is still associated with high mortality [3,4,5,6,7]. The information available on the benefits of va-ECMO is rather inconsistent and scarce [8,9].

Hence, the aim of this work is to explore the overall in-hospital mortality and to identify potential risk factors for mortality. Furthermore, we summarize and compare the clinical and demographic characteristics of patients with cardiogenic shock undergoing ECMO support.

## 2. Materials and Methods

We reviewed the charts of ECMO patients admitted to intensive care units of the Department of Anesthesia and Intensive Care Medicine, Medical University Innsbruck, Austria. The observation period was from 2008 to 2021.

All patients undergoing va-ECMO support were assessed for inclusion in the study. The exclusion criteria were ECMO indications other than cardiogenic shock or cardiac arrest, patients having their second ECMO initiation, support durations of less than six hours, patients younger than ten years, and patients with incomplete datasets. We defined refractory cardiogenic shock as a systolic blood pressure under 90 mmHg or a cardiac index less than 2.2 L/min/m^2^ with inotropic therapy, combined with signs of end-organ perfusion disorders despite the use of all therapeutic support options, such as vasopressors and inotropes [2,10].

We obtained the information on (1) socio-demographic characteristics, (2) indications for ECMO support, (3) critical care prognostic scores as measured by the sequential organ failure assessment (SOFA) and simplified acute physiology III (SAPS III) score on ICU admission, information on mechanical cardiopulmonary reanimation, ECMO support duration, (4) adverse events, (5) use of anticoagulation, and, finally, (6) data on mortality. Two authors independently reviewed the charts and extracted the data.

### 2.1. ECMO Support and Anticoagulation

ECMO support is constantly available in our center. The employed ECMO system consists of a centrifugal pump, unfractionated heparin-coated ECMO circuit, a hollow-fiber oxygenator, and an integrated heat exchanger.

Anticoagulation was conducted according to the institutional standards and based on the anticoagulation recommendations of the ELSO [11,12,13,14]. We used unfractionated heparin (UFH) as the first choice for anticoagulation (targeted activated partial thromboplastin time (aPTT) 50–70 s). In the case of insufficient anticoagulation or a suspected or proven heparin-induced thrombocytopenia type II, argatroban was further utilized. In the presence of severe coagulopathy, continuous anticoagulation was paused. Monitoring and adaptation of anticoagulation therapy were based primarily on the use of aPTT, point-of-care activated clotting time (ACT), blood drug concentration, CT INTEM in the ROTEM^®^, or anti-factor Xa assay activity.

The extracorporeal life support weaning protocol comprised stepwise reduction of flow in the presence of signs of improvement in cardiac function. The heart function was regularly evaluated by using echocardiography. A trial-off was initiated when the ECMO blood flow reached less than 30% of the total and after a joint clinical judgement. ECMO support was terminated in the case of infaust prognosis (due to irreversible heart damage, severe brain damage, irreversible multiple organ failure, etc.). In selected cases, organ explantation and donation were considered.

### 2.2. Outcomes

The primary endpoint was the overall in-hospital mortality of patients receiving va-ECMO support. The secondary endpoints included risk factors and predictors for mortality, comparison of demographic and clinical characteristics in survivors and deceased, and the incidence and types of complications. Finally, we performed subgroup analyses to investigate the outcomes with respect to the ECMO support indication, presence of surgical intervention, and day of ECMO support initiation.

Data on thromboembolic events (type of event, date of identification, and localization) were collected from the charts and radiology findings during the ECMO period, including the two weeks following the cessation of support. Thromboembolic events were diagnosed by using ultrasound or computed tomography. We stratified thrombosis into peripheral thrombus formation (peripheral vein or artery), central venous and arterial thrombosis (i.e., heart, pulmonary artery, aorta, etc.), embolization (i.e., extremities, stroke, etc.), ECMO cannulas and central catheters, and mixed venous and arterial thrombosis.

We collected information on the hemorrhagic complications only during the ECMO support. Any hemorrhage thereafter was not considered as being associated with ECMO. The ELSO definition was used to describe the hemorrhages [11]. A major hemorrhage included a clinically apparent bleeding event with a decrease in hemoglobin (2 g/dL (1.24 mmol/L) or administration of two or more blood concentrates within 24 h). Any retroperitoneal or pulmonary bleeding, bleeding requiring surgical intervention, or bleeding involving the central nervous system was also considered as major. Minor hemorrhages included any other noticeable bleeding [12]. In cases with multiple bleeding events or sources, we analyzed only the date of the first event.

The information on the cause of death was gathered from the available medical documentation or post-mortem examination reports when available. The information on the death date was recorded, and the mortality in different periods was calculated.

We prepared our retrospective work according to the strengthening the reporting of the observational studies in epidemiology (STROBE) statement (Supplementary Table S1) [15].

### 2.3. Statistical Analyses

For the statistical analyses, we used SPSS (Version 28.0. Released 2021, Armonk, NY, USA: IBM Corp.) and the R program (free software for statistical computing and graphics—R Core Team 2020: a language and environment for statistical computing; Vienna, Austria; version 4.0.2). A significance level of 0.05 was employed, and all statistical assessments were two-sided. Depending on the distribution normality and types of variables, we present the results as the mean with the standard deviation, the median with the range (minimum–maximum), and the frequency (percent). An independent-sample *t*-test was used for parametric data, and for numeric data with a non-normal distribution or ordinal data, a Mann–Whitney U or Kruskal–Wallis test was utilized. To test differences among nominal data, we used a chi-square test and Fisher’s exact test. Missing data were not analyzed. Univariate Cox regression analyses were employed to analyze the influences of risk factors on mortality, and all variables with a *p* value under 0.1 were assessed for the multivariate model. The Log Rank test was employed to assess the survival function in its dependence on the ECMO support indication and other variables included in the multivariable model.

## 3. Results

### 3.1. Patient and ECMO Characteristics

After screening all medical records, 453 patients met the inclusion criteria (Table 1 and Table 2). Cardiac surgery was the main reason for ICU admission in 297 (65.6%) patients. The calculated median SAPS III score was 62 (15–104), and the median SOFA score was 11 (1–21).

The median ECMO support duration was six days (Table 2). Anticoagulation was primarily realized with unfractionated heparin or argatroban. Due to life-threatening bleeding or severe coagulopathy, 47 (10%) patients were not under anticoagulation. Finally, 274 (60%) patients were discharged from our hospital.

### 3.2. Outcomes

**Figure 1:**
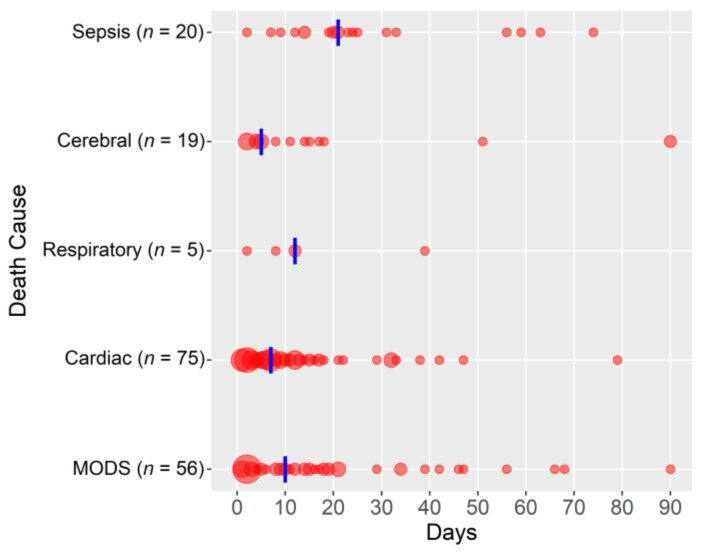
Causes of death (in-hospital mortality; circle size depends on the number of patients; the vertical blue line presents the median; *n* = 175, missing data: 4/453). Abbreviations: MODS, multiple organ dysfunction syndrome.

The overall in-hospital all-cause mortality was 40% (179/453), and cardiac failure and multiple organ dysfunction syndrome were the main causes of death (Figure 1). The Kaplan–Meier mean in-hospital survival estimate was 60.7 days (95%CI 57.2–64.2, Supplementary Figure S1). A comparison of patients based on the cause of cardiogenic shock and in-hospital mortality and the Kaplan–Meier estimates are presented in Supplementary Figures S2–S9.

Patients who were deceased had significantly higher SOFA and SAPS III scores, had a shorter length of ICU stay, and were more often reanimated (Table 1). The most frequent complication was hemorrhage (209, 46%), which occurred on the second day of ECMO. Moreover, deceased patients experienced more bleeding events (Table 2).

The univariate analyses identified a higher SOFA and SAPS III score, a cardiac and non-surgical cause of cardiogenic shock, cardiopulmonary reanimation before or during initiation of ECMO support, and initiation on weekends as independent predictors for in-hospital mortality. Among the adverse events, hemorrhage and sepsis were associated with an increased mortality risk (Supplementary Table S2). Finally, the multivariate model identified hemorrhage, initiation of ECMO on weekends, a higher SAPS III score, and sepsis as having an increased hazard ratio for mortality (Table 3).

### 3.3. Subgroup Analyses

Postcardiotomy patients were older, had lower SAPS III scores, were resuscitated less often, received ECMO support less often on weekends, and had lower in-hospital mortality (Supplementary Table S3). Furthermore, the initiation of ECMO support on weekends was associated with a higher ICU, in-hospital, and one-year mortality (Supplementary Table S4). The analysis of the ECMO indications showed that patients with chronic heart failure had the lowest mortality (Supplementary Table S5). Conversely, 52% of patients with an acute heart failure were resuscitated before or during the initiation of ECMO and had the highest mortality.

## 4. Discussion

This study is one of the largest retrospective reports on risk factors for mortality during va-ECMO support. Deceased patients (1) were sicker, (2) were resuscitated more often, and (3) received ECMO more frequently on weekends. Moreover, the ECMO course was often complicated by hemorrhage and sepsis. Finally, nearly two-thirds of the patients with refractory cardiogenic shock survived.

Our findings on one-year survival are comparable to those of a meta-analysis reporting a nearly 54% survival rate for patients receiving va-ECMO for cardiogenic shock and cardiac arrest [8]. In the etiology of cardiogenic shock, patients with chronic heart failure were the youngest, had the lowest SAPS III and SOFA scores, and had the longest ICU stays. This group of patients had the lowest mortality, and most of them received ECMO support as a bridge to permanent assistance or transplantation according to the guidelines of the European Society of Cardiology [16]. Conversely, patients with an acute heart failure were rather young, but with higher SAPS III scores, and more than half of them were mechanically resuscitated. These patients had the highest mortality in all observed periods, but this rate was still within the range reported in a meta-analysis comprising 10,276 patients with acute heart failure and was lower compared to the pooled mortality estimate of 58% [17].

Finally, regarding adverse events, hemorrhage occurred most often in the group of patients who underwent the combined procedures of coronary artery bypass and heart valve surgery. Thrombosis ensued most often in patients without cardiotomy, and sepsis complicated the course of procedures for treating chronic heart failure. The reporting on adverse events during ECMO support is limited by the diversity of reporting in published studies and the high heterogeneity in the available meta-analyses, making any comparison complex. A list of reporting items and definitions of adverse events is warranted in the future.

### 4.1. Risk Factors for Mortality

Only a handful of studies focused on predictors and risk factors of mortality during va-ECMO support [8,9,17,18,19]. Our univariate analyses identified higher SOFA and SAPS III scores, a cardiac and non-surgical cause of cardiogenic shock, resuscitation, initiation on weekends, hemorrhage, and sepsis as independent predictors of in-hospital mortality. The multivariate model confirmed hemorrhage, a higher SAPS III score, initiation of support on weekends, and sepsis as variables with increased hazard ratios for in-hospital mortality.

Interestingly, older age and presence of thromboembolism were not associated with increased mortality. The role of age is still a subject of debate, and clear recommendations for va-ECMO are not available. However, in the case of COVID-19, the ELSO defined the age of 65 as a relative contraindication for ECMO support and advanced age as an absolute contraindication, leaving the definition of advanced age open [20]. On the other hand, the evidence on the impact of thromboembolic complications on the survival of patients receiving va-ECMO is sparse. The true incidence of thromboembolic events is still unknown, as their diagnosis may often be missed due to the lack of clinical manifestations. In a post-mortem evaluation of almost 80 postcardiotomy ECMO patients, only one-third of the 36 patients experiencing a thromboembolic event were diagnosed ex ante [21]. Moreover, only a few studies reported on the incidence of thrombosis, which ranged between 16% and 46% [21,22,23,24]. Further research on the roles of thromboembolic complications and va-ECMO outcomes is warranted.

The accuracy and use of prognostic scores remain subjects of dispute. We identified higher SOFA and SAPS III scores as predictors of an increased in-hospital mortality. Consistently with the literature, deceased patients had a SAPS III score of 71 points, which was 13 points higher than that of survivors [25,26]. The information on prognostic scores for extracorporeal life support patients and the reporting on these scores are rather sparse and heterogeneous, thus limiting comparisons. Most publications missed the inclusion of the SAPS III score in their reports, and only a few concluded that other scores may be potentially associated with mortality [18,27]. There is not a specific prognostic score cut-off, but there is a list of relative contraindications provided by the ELSO, such as futility and nonrecoverable comorbidity, risk of systemic bleeding with anticoagulation, etc. [2]. The decision on the initiation of ECMO support should rather be based on the severity of the disease in combination with assessment of the frailty, comorbidities, and rehabilitation potential of the patient, warranting individualized risk–benefit considerations for every patient.

In deceased patients, ECMO support was started more often on weekends. The “weekend effect” is often reported in the literature [28,29]. We found that patients receiving ECMO support on weekends had higher SAPS III scores, were mechanically resuscitated more often, and underwent cardiac surgery less often. The higher SAPS III score could be explained by emergency procedures (increasing the score by five points). The lower incidence of cardiac surgery on weekends may be associated with the main etiology (acute heart failure in 42%) or the initiation of ECMO under resuscitation (37% on weekends vs. 26% on working days). Finally, patients receiving ECMO support on weekends experienced hemorrhage less often. This could be explained by the lower incidence of cardiac surgery (Supplementary Table S4).

Finally, patients with bleeding had almost two times higher chance of death, making it a main risk factor for in-hospital mortality. Bleeding is still the most serious and common adverse event, and it has the potential for severe invalidity, permanent injury, or even death [30]. In our study population, nearly 50% of the patients experienced bleeding events, of which 57% had major bleeding events. Deceased patients experienced bleeding events more often. The available literature from both main ECMO configurations found that bleeding is associated with worse patient outcomes [30,31,32,33,34,35]. However, only a few studies have reported on hemorrhage during va-ECMO, and they have rarely used the ELSO definition. The cumulative overall incidence of bleeding in the case of va-ECMO described in a recent systematic review was 33%, with 28% experiencing severe or major bleeding [36]. In contrast to that, a meta-analysis of patients with cardiogenic shock or cardiac arrest reported on a pooled estimated rate of major or significant bleeding being 41% (95% CI 26.8–56.6%). However, this was limited by the inclusion of only five studies and 260 patients [19]. The varying incidences reported above may be explained by the rather sparse evidence on bleeding during va-ECMO support, the heterogeneity in reporting, and the different definitions of bleeding. Despite the existence of the ELSO’s definition of bleeding for more than eight years [11], its utilization is rather rare, making any comparison complex.

Both sepsis and higher SOFA scores were associated with increased risk of mortality, presenting poor prognostic markers for patients undergoing ECMO support. There have been reports of successful utilization of ECMO as a rescue option for cardiogenic shock induced by sepsis, but it is still characterized by a high risk of death [18,37,38]. In our study, 24% of the deceased patients experienced sepsis during ECMO support, and 20 patients died due to sepsis or septic shock. Therefore, continuous monitoring, early clinical suspicion, early diagnosis, and commencement of antimicrobial treatment may considerably influence mortality due to sepsis during ECMO support [32,37,39].

### 4.2. Limitations

This study has certain limitations. As in any retrospective study, a selection bias cannot be excluded. Moreover, we excluded patients with an ECMO duration of less than six hours (*n* = 18), as well as patients with incomplete datasets (*n* = 3). This may have led to a further selection bias. However, the number of excluded patients was rather small. We observed outcomes of ECMO patients over a period of 14 years. As evidence on advanced heart failure has evolved in the meantime, with some new treatments appearing, this may entail a potential limitation. It is complex to distinguish potential complications of an underlying illness from adverse ECMO-related events. However, the reported hemorrhages were most probably a consequence of both distorted coagulation and ECMO support. Some adverse events may have been missed if additional diagnostic procedures had not been performed. The diagnosis of thrombosis was only possible with the available radiological investigations or post-mortem examinations, which may have led to its underestimation. However, to identify as many thrombotic events that were potentially ECMO-associated as possible, we examined all radiological reports during ECMO support and within two weeks of its termination. Lastly, in the absence of randomized clinical trials, retrospective analyses of patients’ data present a basis for meta-analyses and a summarization of the evidence. Here, we provide additional information on predictors and risk factors for mortality in patients with cardiogenic shock who have received ECMO support.

## 5. Conclusions

In this retrospective study from a European university center, 40% of patients died during the hospital stay. We identified hemorrhage, a higher SAPS III score, initiation on weekends, and sepsis as significant risk factors for mortality. Unfavorable events during ECMO support are common and can have potential for permanent injury or death. Hemorrhage was the most common complication, with major bleeding events dominating in deceased patients. Although the rates of complications are substantial, a well-selected proportion of patients with refractory cardiogenic shock may be rescued from probable death. Finally, the reported risk factors may be used to increase the awareness of researchers and clinicians towards the development of new therapeutic concepts that could reduce their incidence.

## Figures and Tables

**Table 1:** Characteristics of the included patients (*n* = 453, in-hospital mortality).

Patient Characteristics	All Patients (*n* = 453)	Survivors (*n* = 274)	Non-Survivors (*n* = 179)	*p*-Value	Missing Data (*n*/Total)
Age (years)	60.9 ± 14.2	60.1 ± 14.1	62.0 ± 14.3	0.165	0/453
<30	18 (4.0)	11 (4.0)	7 (3.9)	0.198	0/453
31–45	41 (9.1)	27 (9.9)	14 (7.8)		
46–60	140 (30.9)	94 (34.3)	46 (25.7)		
61–75	190 (41.9)	109 (39.8)	81 (45.3)		
>75	64 (14.1)	33 (12.0)	31 (17.3)		
Male sex	314 (69.3)	193 (70.4)	121 (67.6)	0.533	0/453
Body mass index (kg/m^2^)	26.5 ± 4.6	26.4 ± 4.8	26.8 ± 4.3	0.294	5/453
SAPS III score	62 (15–104)	58 (15–99)	71 (31–104)	<0.001	1/453
SAPS III score predicted mortality (%)	40 (0–93)	32 (0–91)	58 (2–93)	<0.001	1/453
SOFA score	11 (1–21)	11 (2–21)	12 (1–20)	0.006	1/453
SOFA respiratory	2 (0–4)	2 (0–4)	2 (0–4)	0.529	
SOFA coagulation	1 (0–4)	1 (0–3)	1 (0–4)	0.322	
SOFA liver	1 (0–4)	1 (0–3)	0 (0–4)	0.539	
SOFA cardiovascular	4 (0–4)	4 (0–4)	4 (0–4)	<0.001	
SOFA neurology	4 (0–4)	4 (0–4)	4 (0–4)	0.024	
SOFA renal	1 (0–4)	1 (0–4)	1 (0–4)	0.013	
CPR before ECMO initiation	127 (28.0)	63 (23.0)	64 (35.8)	0.004	0/453
Length of ICU stay (days)	17 (1–170)	21 (4–98)	10 (1–170)	<0.001	0/453
Cardiogenic shock etiology					0/453
No cardiotomy					
Acute heart failure	126 (27.8)	63 (23.0)	63 (35.2)	0.007	
Right heart failure	30 (6.6)	16 (5.8)	14 (7.8)		
Postcardiotomy					
Coronary artery bypass surgery (CABG)	66 (14.6)	35 (12.8)	31 (17.3)		
Heart valve surgery (HVS)	164 (36.2)	113 (41.2)	51 (28.5)		
Combined (CABG and HVS, including aortic aneurysm)	43 (9.5)	29 (10.6)	14 (7.8)		
Chronic heart failure	24 (5.3)	18 (6.6)	6 (3.4)		
Mortality-related outcomes					0/453
Admission to death (days)	10 (1–79)	-	-		
ECMO initiation to death (days)	9 (1–79)	-	-		
Death during ECMO support	86 (19.0)	-	-		
Death during ICU stay	165 (36.4)	-	-		
Death within 30 days	150 (33.1)	-	-		
Death within 90 days	178 (39.3)	-	-		
Death within 180 days	183 (40.4)	-	-		
Death within 365 days	185 (40.8)	-	-		
Survival beyond one year	268 (59.2)	-	-		

Data are presented as the number of patients (%), mean ± standard deviation, or median (minimum–maximum). Abbreviations: ECMO, extracorporeal membrane oxygenation; ICU, intensive care unit; CPR, cardiopulmonary resuscitation; SAPS III, simplified acute physiology score III; SOFA, sequential organ failure assessment score.

**Table 2:** ECMO support characteristics and patient outcomes (*n* = 453, in-hospital mortality).

Characteristics	All Patients (*n* = 453)	Survivors (*n* = 274)	Non-Survivors (*n* = 179)	*p*-Value	Missing Data (*n*/Total)
Postcardiotomy					0/453
No cardiotomy	156 (34.4)	79 (28.8)	77 (43.0)	0.002	
Postcardiotomy	297 (65.6)	195 (71.2)	102 (57.0)		
ECMO course					0/453
ECMO duration (days)	6 (1–22)	6 (1–20)	6 (1–22)	0.364	
ECMO duration <7 days	326 (72.0)	204 (74.5)	122 (68.2)	0.164	
Admission to ECMO initiation (days)	0 (0–20)	0 (0–15)	0 (0–20)	0.747	
Day of ECMO initiation					0/453
Weekday	355 (78.4)	224 (81.8)	131 (73.2)	0.036	
Weekends	98 (21.6)	50 (18.2)	48 (26.8)		
Anticoagulation during ECMO support					0/453
UFH	320 (70.6)	200 (73.0)	120 (67.0)	0.002	
Argatroban	72 (15.9)	46 (16.8)	26 (14.5)		
Epoprostenol	2 (0.4)	0 (0.0)	2 (1.1)		
UFH switch to argatroban	12 (2.6)	10 (3.6)	2 (1.1)		
None	47 (10.4)	18 (6.6)	29 (16.2)		
Reason for ECMO cessation					0/453
Improvement (weaned)	328 (72.4)	247 (90.1)	81 (45.3)	<0.001	
Bridge to other assistance (heart transplant or VAD)	32 (7.1)	26 (9.5)	6 (3.4)		
Hemorrhage	7 (1.5)	1 (0.4)	6 (3.4)		
Death	86 (19.0)	-	86 (48.0)		
Complications					
Hemorrhage	209 (46.1)	107 (39.1)	102 (57.0)	<0.001	0/453
Major hemorrhage	119 (26.3)	56 (20.4)	63 (35.2)	0.001	0/453
Minor hemorrhage	90 (19.9)	51 (18.6)	39 (21.8)	0.470	0/453
Hemorrhage day	2 (1–14)	2 (1–14)	2 (1–14)	0.578	0/453
Hemorrhage within first three days	150 (74.3)	76 (73.8)	74 (74.7)	1.000	0/453
Thrombosis	114 (25.2)	71 (25.9)	43 (24.0)	0.740	0/453
Thrombosis venous	68 (15.0)	52 (19.0)	16 (8.9)	0.003	0/453
Thrombosis arterial	68 (11.2)	31 (11.3)	37 (20.7)	0.007	0/453
Sepsis	83 (18.4)	41 (15.0)	42 (23.6)	0.025	0/453

Data are presented as the number of patients (%), median (minimum–maximum), or mean ± standard deviation. Abbreviations: ECMO, extracorporeal membrane oxygenation; UFH, unfractionated heparin; VAD, ventricular assistance device.

**Table 3:** Identification of risk factors for mortality (multivariate analysis; *n* = 453).

Variable	B-Coefficient	*p*-Value	HR	95% Confidence Interval	
				Lower	Upper
Hemorrhage	0.542	0.001	1.72	1.26	2.34
SAPS III score	0.030	<0.001	1.03	1.02	1.04
ECMO initiation (weekend)	0.383	0.028	1.47	1.04	2.06
Sepsis	0.381	0.032	1.46	1.03	2.07
Resuscitation before ECMO	0.256	0.125	1.29	0.93	1.79
No cardiotomy	0.149	0.362	1.16	0.84	1.60

Abbreviations: CI, confidence interval; HR, hazard ratio; ECMO, extracorporeal membrane oxygenation; SAPS III, simplified acute physiology score III (cases with missing data: 2/453).

## Data Availability

The datasets used and analyzed in the current study are made available from the corresponding author on reasonable request.

## Supplementary Materials

The following supporting information can be downloaded at: https://www.mdpi.com/article/10.3390/jcm11226821/s1, Table S1. STROBE Statement—Checklist of items that should be included in reports of cohort studies; Figure S1. Kaplan–Meier estimate of all-cause in-hospital mortality; Figure S2. Comparison of patients based on the cause of cardiogenic shock and in-hospital mortality; Figure S3. Kaplan–Meier mean estimate of all-cause in-hospital mortality: ECMO indication; Figure S4. Kaplan-Meier mean survival estimate: presence of cardiotomy; Figure S5. Kaplan–Meier mean survival estimate with and without hemorrhage; Figure S6. Kaplan–Meier mean survival estimate: the day of ECMO initiation; Figure S7. Kaplan–Meier mean survival estimate: sepsis; Figure S8. Kaplan–Meier mean survival estimate: resuscitation before ECMO; Figure S9. Kaplan-–Meier mean survival estimate: the SAPS III score; Table S2. Subgroup analysis: Demographic and clinical characteristics of patients with cardiogenic shock and the presence of postcardiotomy; Table S3. Subgroup analysis: ECMO initiation on working days or weekends; demographic and clinical characteristics; Table S4. Risk factors for in-hospital mortality: univariate Cox regression analyses; Table S5. Selected demographic and clinical characteristics of patients according to the cause of cardiogenic shock.
