# Supplementary material for: ECMO Support in Refractory Cardiogenic Shock: Risk Factors for Mortality
Source: J Clin Med. 2022 Nov 18;11(22):6821. doi: 10.3390/jcm11226821 (PMC9698852; doi:10.3390/jcm11226821)
Supplement: Supplementary file 1 [file jcm-11-06821-s001.zip › jcm-2016006-supplementary - final version (1).pdf]

Journal of Clinical Medicine

MDPI

Article

ECMO support in Refractory Cardiogenic Shock: Risk Factors for Mortality

Sasa Rajsic, Robert Breitkopf, Zoran Bukumirić and Benedikt Treml

Supplementary material

Table S1. STROBE Statement - Checklist of items that should be included in reports of cohort studies
Figure S1. Kaplan-Meier mean survival estimate (*n* = 453)
Figure S2. Comparison of patients based on the cause of cardiogenic shock and in-hospital mortality (*n* = 453)
Figure S3. Kaplan-Meier mean survival estimate: ECMO indication
Figure S4. Kaplan-Meier mean survival estimate: cardiotomy presence
Figure S5. Kaplan-Meier mean survival estimate with and without haemorrhage
Figure S6. Kaplan-Meier mean survival estimate: the day of ECMO initiation
Figure S7. Kaplan-Meier mean survival estimate: sepsis
Figure S8. Kaplan-Meier mean survival estimate: resuscitation before ECMO
Figure S9. Kaplan-Meier mean survival estimate: the SAPS III score
Table S2. Subgroup analysis: Demographic and clinical characteristics of patients with cardiogenic shock and the presence of postcardiotomy (*n* = 453)
Table S3. Subgroup analysis: ECMO initiation on working days or weekends; demographic and clinical characteristics (*n* = 453)
Table S4. Risk factors for in-hospital mortality: univariate Cox regression analyses (*n* = 453)
Table S5. Selected demographic and clinical characteristics of patients according to the cause of cardiogenic shock (*n* = 453)

Journal of Clinical Medicine

Table S1. STROBE Statement - Checklist of items that should be included in reports of cohort studies.

No.	Item	Recommendation	Page
	**Title and abstract**		
1		(*a*) Indicate the study's design with a commonly used term in the title or the abstract	2
		(*b*) Provide in the abstract an informative and balanced summary of what was done and what was found	
	Introduction		
2	Background/rationale	Explain the scientific background and rationale for the investigation being reported	3
3	Objectives	State specific objectives, including any prespecified hypotheses	
	Methods		
4	Study design	Present key elements of study design early in the paper	3–4
5	Setting	Describe the setting, locations, and relevant dates, including periods of recruitment, exposure, follow-up, and data collection	3–4
6	Participants	(*a*) Give the eligibility criteria, and the sources and methods of selection of participants. Describe methods of follow-up	3–4
		(*b*) For matched studies, give matching criteria and number of exposed and unexposed	3–4
7	Variables	Clearly define all outcomes, exposures, predictors, potential confounders, and effect modifiers. Give diagnostic criteria, if applicable	3–4
8*	Data sources/ measurement	For each variable of interest, give sources of data and details of methods of assessment (measurement). Describe comparability of assessment methods if there is more than one group	*Online resource*
9	Bias	Describe any efforts to address potential sources of bias	11
10	Study size	Explain how the study size was arrived at	NA
11	Quantitative variables	Explain how quantitative variables were handled in the analyses. If applicable, describe which groupings were chosen and why	4
12	Statistical methods	(*a*) Describe all statistical methods, including those used to control for confounding	4
		(*b*) Describe any methods used to examine subgroups and interactions	4
		(*c*) Explain how missing data were addressed	4
		(*d*) If applicable, explain how loss to follow-up was addressed	4
		(*e*) Describe any sensitivity analyses	4
	Results		
13*	Participants	(a) Report numbers of individuals at each stage of study—eg numbers potentially eligible, examined for eligibility, confirmed eligible, included in the study, completing follow-up, and analysed	4
		(b) Give reasons for non-participation at each stage	4
		(c) Consider use of a flow diagram	
14*	Descriptive data	(a) Give characteristics of study participants (eg demographic, clinical, social) and information on exposures and potential confounders	4–5
		(b) Indicate number of participants with missing data for each variable of interest	4–7
		(c) Summarise follow-up time (eg, average and total amount)	4
15*	Outcome data	Report numbers of outcome events or summary measures over time	4–7
16	Main results	(*a*) Give unadjusted estimates and, if applicable, confounder-adjusted estimates and their precision (eg, 95% confidence interval). Make clear which confounders were adjusted for and why they were included	4–7
		(*b*) Report category boundaries when continuous variables were categorized	4–7

		(*c*) If relevant, consider translating estimates of relative risk into absolute risk for a meaningful time period	
17	Other analyses	Report other analyses done—eg analyses of subgroups and interactions, and sensitivity analyses	8
	Discussion		
18	Key results	Summarise key results with reference to study objectives	8–11
19	Limitations	Discuss limitations of the study, taking into account sources of potential bias or imprecision. Discuss both direction and magnitude of any potential bias	11
20	Interpretation	Give a cautious overall interpretation of results considering objectives, limitations, multiplicity of analyses, results from similar studies, and other relevant evidence	11
21	Generalisability	Discuss the generalisability (external validity) of the study results	8–11
	Other information		
22	Funding	Give the source of funding and the role of the funders for the present study and, if applicable, for the original study on which the present article is based	Title page

*Give information separately for exposed and unexposed groups.

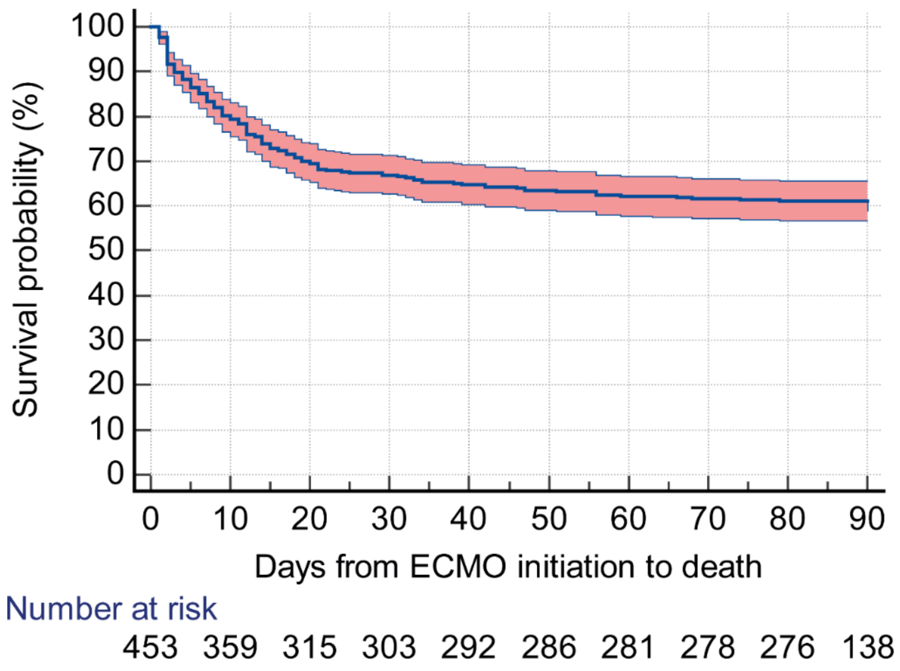

Figure S1. Kaplan-Meier mean survival estimate (n = 453, mean 60.7, 95% CI 57.2 - 64.2).

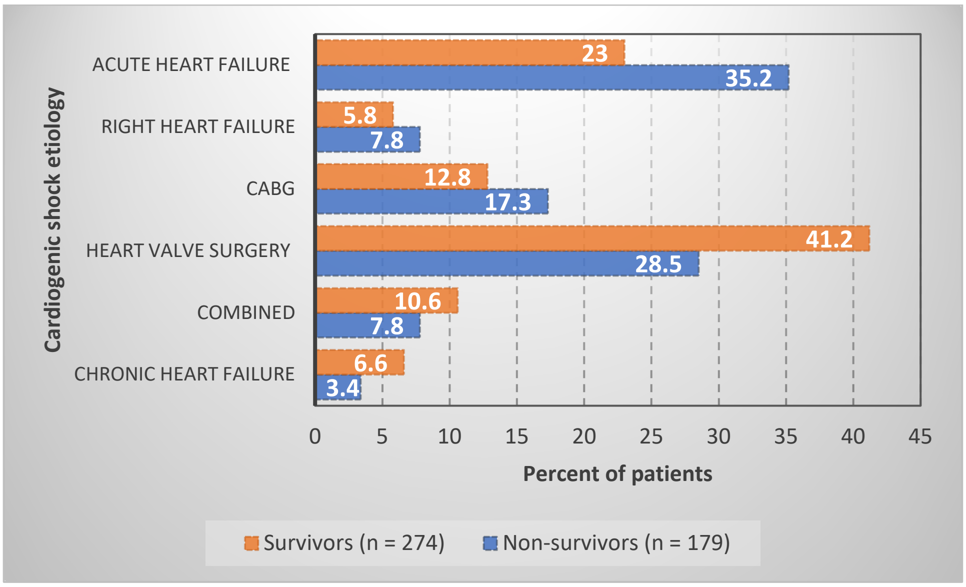

Figure S2. Comparison of patients based on the cause of cardiogenic shock and in-hospital mortality (n = 453). Red colour: survivors, n = 274; blue colour: non-survivors, n = 179. Abbreviations: CABG, Coronary artery bypass graft.

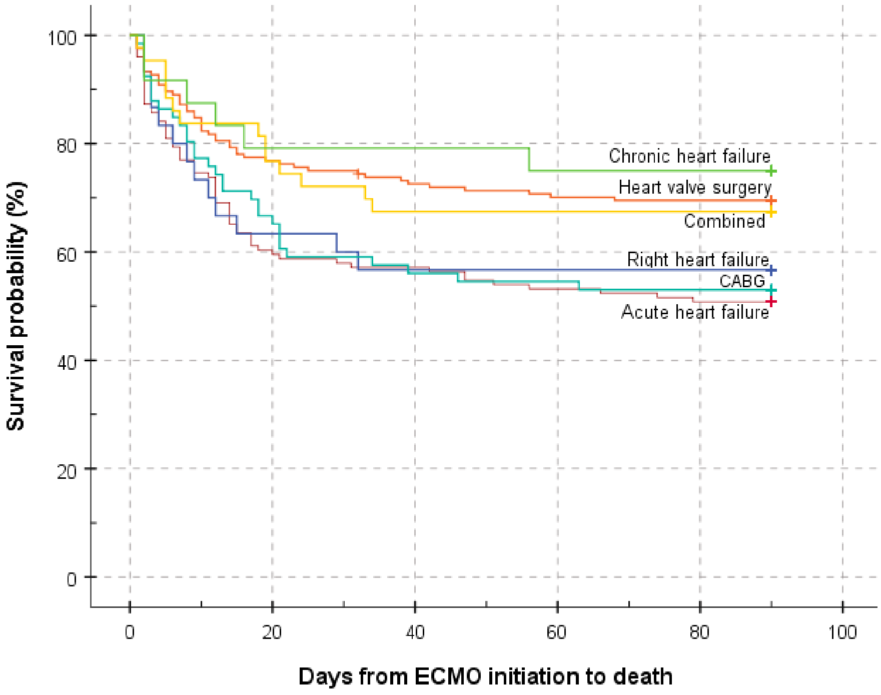

Figure S3. Kaplan-Meier mean survival estimate: ECMO indication (***n*** = 453). Abbreviations: ECMO, extracorporeal membrane oxygenation; CABG, coronary artery bypass surgery; CI: confidence intervals.

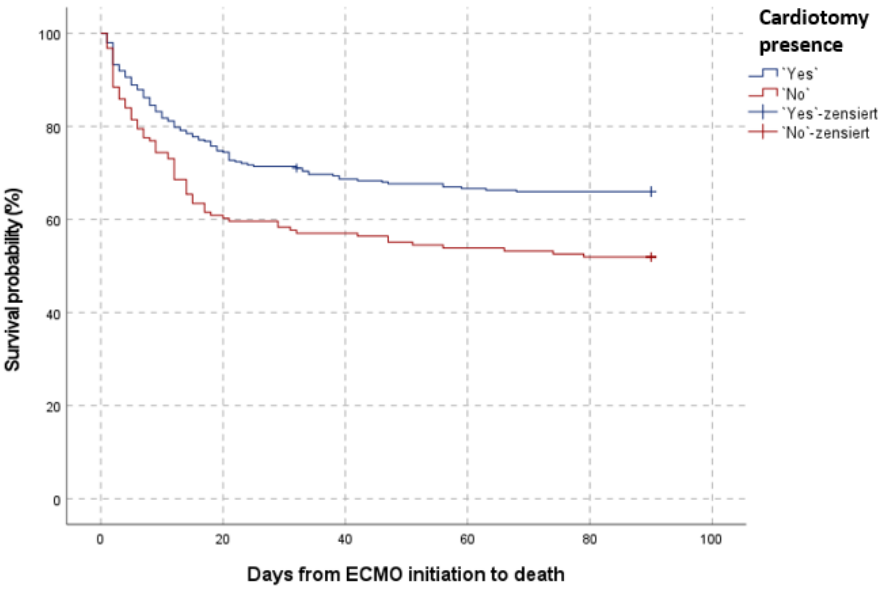

Figure S4. Kaplan-Meier mean survival estimate: cardiotomy presence (n = 453). Mean estimate 65 days for postcardiotomy patients (n = 297; 95% CI 60.3 - 68.6) and 54 days for patients without surgical intervention (n = 156; 95% CI 47.4 - 59.8). Abbreviations: ECMO, extracorporeal membrane oxygenation; CI: confidence intervals.

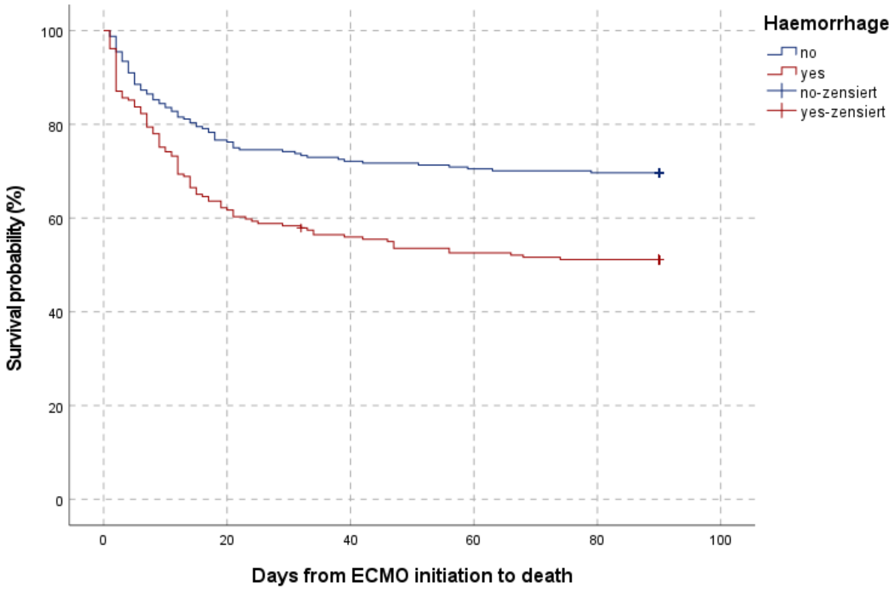

Figure S5. Kaplan-Meier mean survival estimate with and without haemorrhage (n = 453). Mean estimate 53 days for patients with bleeding event (n = 209; 95% CI 47.9 - 58.5) and 67 days for patients without bleeding event (n = 244; 95% CI 62.7 - 71.7). Abbreviations: ECMO, extracorporeal membrane oxygenation; CI: confidence intervals.

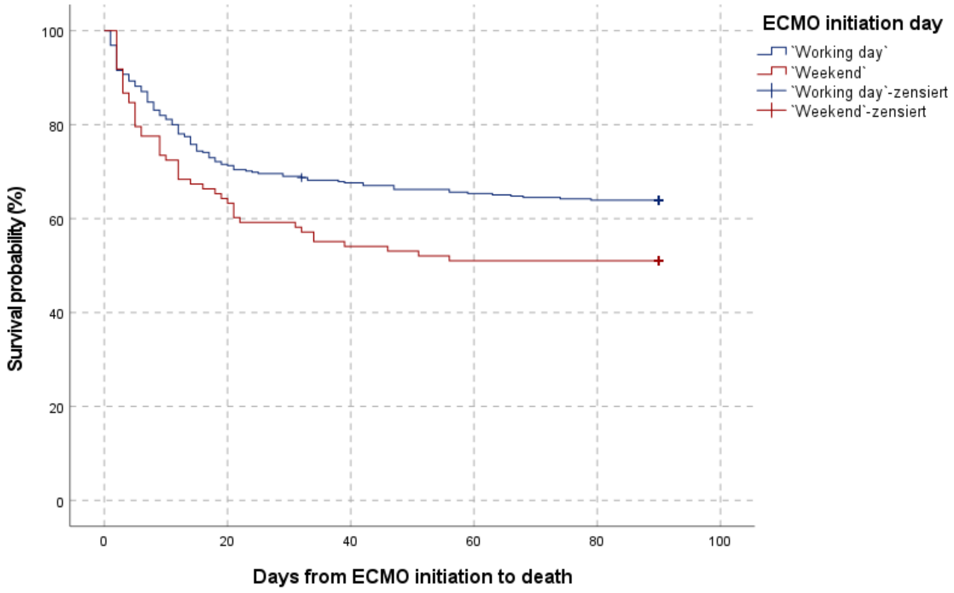

Figure S6. Kaplan-Meier mean survival estimate: the day of ECMO initiation (*n* = 453). Mean estimate 53 days for patients with ECMO support initiation on weekend (*n* = 98; 95% CI 44.9 - 60.5) and 63 days for patients with ECMO support initiation on working days (*n* = 355; 95% CI 59.0 - 66.8). Abbreviations: ECMO, extracorporeal membrane oxygenation; CI: confidence intervals.

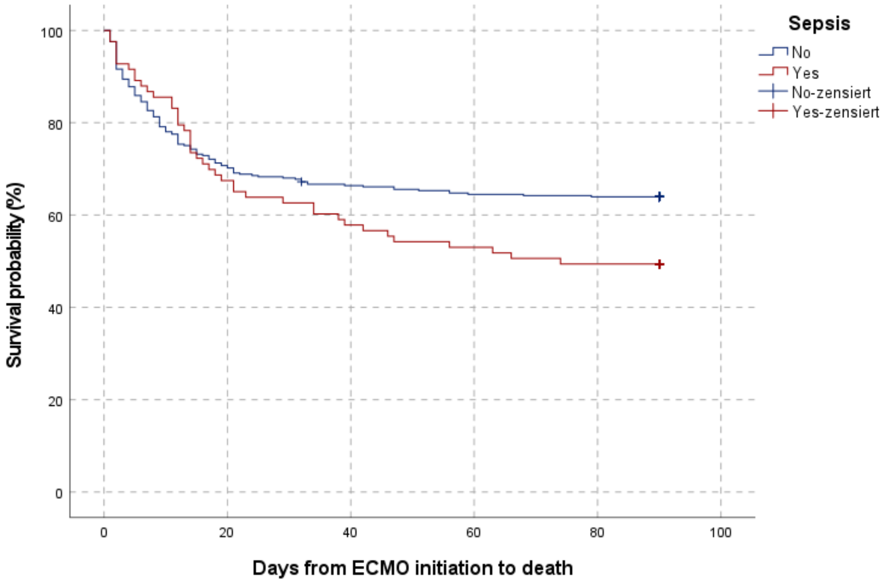

Figure S7. Kaplan-Meier mean survival estimate: sepsis (*n* = 453). Mean estimate 55 days for patients with sepsis (*n* = 83; 95% CI 47.2 - 63.1) and 62 days for patients without sepsis (*n* = 370; 95% CI 58.2 - 66.0). Abbreviations: ECMO, extracorporeal membrane oxygenation; CI: confidence intervals.

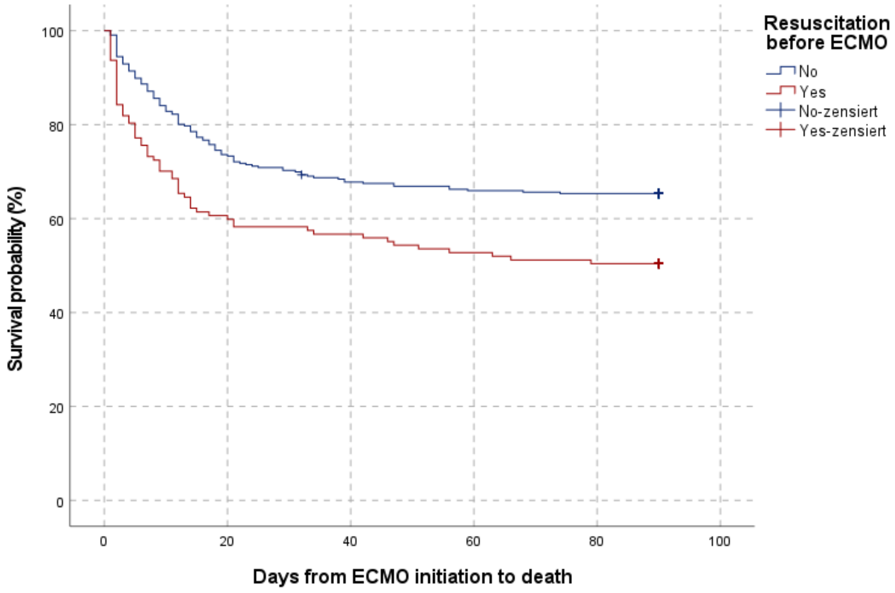

Figure S8. Kaplan-Meier mean survival estimate: resuscitation before ECMO (*n* = 453). Mean estimate 52 days for patients who were resuscitated (*n* = 127; 95% CI 45.3 - 59.2) and 64 days for patients who were not resuscitated (*n* = 326; 95% CI 60.1 - 68.0). Abbreviations: ECMO, extracorporeal membrane oxygenation; CI: confidence intervals.

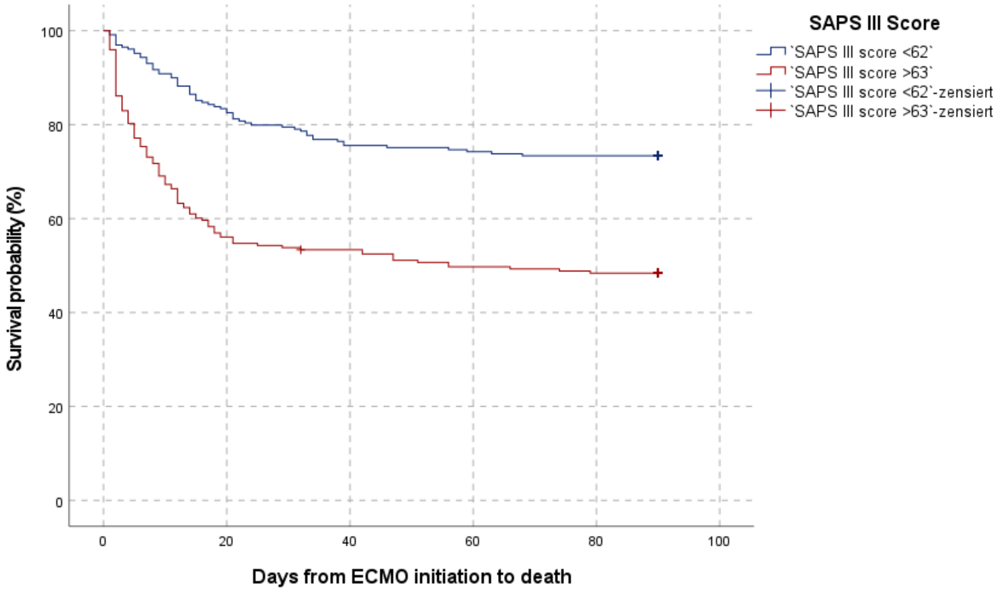

Figure S9. Kaplan-Meier mean survival estimate: the SAPS III score (*n* = 452). Mean estimate 71 days for patients with SAPS III score under 62 (*n* = 229; 95% CI 66.8 - 75.3) and 50 days for patients with SAPS III score above 63 (*n* = 223; 95% CI 44.7 - 55.3). Abbreviations: ECMO, extracorporeal membrane oxygenation; CI: confidence intervals; SAPS III: simplified acute physiology score III.

Table S2. Risk factors for in-hospital mortality: univariate Cox regression analyses (n = 453).

Nondependent variable	B-coefficient	p-value	HR	95% confidence interval	
				lower	upper
Age (years)	0.010	0.085	1.01	1.00	1.02
Sex (male/female)	0.083	0.608	1.09	0.79	1.49
Height (cm)	−1.191	0.144	0.30	0.06	1.50
Weight (kg)	0.000	0.967	1.00	1.00	1.01
Body mass index (kg/m²)	0.016	0.332	1.02	0.98	1.05
SAPS III score	0.034	<0.001	1.04	1.03	1.04
SOFA Score	0.061	0.002	1.06	1.02	1.11
Reanimation before ECMO	0.508	0.001	1.66	1.22	2.26
ICU length of stay	−0.070	<0.001	0.93	0.92	0.95
Presence of cardiac surgery	0.457	0.003	1.58	1.17	2.13
ECMO duration	−0.029	0.205	0.97	0.93	1.02
ECMO initiation on weekend	0.390	0.021	1.48	1.06	2.06
ECMO support indication (reference category acute heart failure)					
Right heart failure	−0.609	0.001	0.54	0.38	0.79
Coronary artery bypass surgery (CABG)	−0.546	0.065	0.58	0.32	1.04
Heart valve surgery (HVS)	−0.088	0.691	0.92	0.60	1.41
Combined (CABG and HVS, including aortic aneurysm)	−0.844	0.048	0.43	0.19	0.99
Chronic heart failure	−0.145	0.635	0.87	0.48	1.57
Thrombosis	−0.048	0.785	0.95	0.68	1.35
Hemorrhage	0.602	<0.001	1.83	1.35	2.46
Major hemorrhage	0.598	<0.001	1.82	1.34	2.48
Minor hemorrhage	0.159	0.382	1.17	0.82	1.67
Sepsis	0.344	0.052	1.41	1.00	2.00
C-reactive protein (mg/dL)	0.014	0.170	1.01	0.99	1.04
Procalcitonin (µg/L)	−0.010	0.472	0.99	0.96	1.02

Abbreviations: CI, confidence Intervals; HR, hazard ratio; SAPS III, simplified acute physiology score III; SOFA, sequential organ failure assessment score; ICU, intensive care unit; ECMO, extracorporeal membrane oxygenation.

Table S3. Subgroup analysis: Demographic and clinical characteristics of patients with cardiogenic shock and presence of postcardiotomy (n = 453).

Characteristics	Postcardiotomy (n = 297)	No cardiotomy (n = 156)	p-value	Missing data (n/total)
Age (years)	63.1 ±14.1	56.6 ±13.4	<0.001	0/453
Male sex	201 (67.7)	113 (72.4)	0.335	0/453
Body mass index (kg/m²)	26.4 ±4.7	26.9 ±4.4	0.251	5/453
SOFA score	11 (2–20)	11 (1–21)	0.375	1/453
SAPS III Score	58 (15–101)	68 (37–104)	<0.001	1/453
CPR before ECMO initiation	55 (18.5)	72 (46.2)	<0.001	0/453
ECMO duration (days)	6 (1–22)	6 (1–17)	0.482	0/453
ICU length of stay (days)	18 (1–170)	14 (1–121)	0.009	0/453
ECMO initiation on weekend	54 (18.2)	44 (28.2)	0.016	0/453
Anticoagulation during ECMO support				0/453
UFH	212 (71.4)	108 (69.2)	0.517	
Argatroban	42 (14.1)	30 (19.2)		
Epoprostenol	1 (0.3)	1 (0.6)		
UFH switch to argatroban	8 (2.7)	4 (2.6)		
None	34 (11.4)	13 (8.3)		
Reason for ECMO support termination				0/453
Improvement	229 (77.1)	99 (63.5)	0.016	
Death	45 (15.2)	41 (26.3)		
Successful bridging	19 (6.4)	13 (8.3)		
Haemorrhage	4 (1.3)	3 (1.9)		
Complications				0/453
Haemorrhage	134 (45.1)	75 (48.1)	0.554	
Major haemorrhage	96 (32.3)	23 (14.7)	<0.001	
Minor haemorrhage	38 (12.8)	52 (33.3)	<0.001	
Thrombosis	70 (23.6)	44 (28.2)	0.306	
Sepsis	54 (18.2)	29 (18.6)	1.000	
Mortality-related outcomes				0/453
Death on ECMO	45 (15.2)	41 (26.3)	0.005	
ICU-mortality	95 (32.0)	70 (44.9)	0.008	
In-hospital mortality	102 (34.3)	77 (49.4)	0.002	
1-year mortality	105 (35.4)	80 (51.3)	0.001	
Cause of death				4/453
Multiple organ failure	37 (35.9)	19 (26.4)	0.101	
Cardiac cause	41 (39.8)	34 (47.2)		
Respiratory failure	3 (2.9)	2 (2.8)		
Sepsis	15 (14.6)	5 (6.9)		
Brain death	7 (6.8)	12 (16.7)		

Data presented as mean ± standard deviation, median (minimum – maximum range) or number of patients (%). Abbreviations: SAPS III, simplified acute physiology score III; SOFA, sequential organ failure assessment score; ICU, intensive care unit; ECMO, extracorporeal membrane oxygenation; CPR, cardiopulmonary resuscitation, UFH, Unfractionated heparin.

Table S4. Subgroup analysis: ECMO initiation on working days or weekends; demographic and clinical characteristics (n = 453).

Characteristics	Working day (n = 355)	Weekend (n = 98)	p-value	Missing data (n/total)
Age (years)	61.4 ±14.1	58.9 ±14.4	0.135	0/453
Male sex	241 (67.9)	73 (74.5)	0.219	0/453
Body mass index (kg/m²)	26.6 ±4.6	26.3 ±4.5	0.574	5/453
SOFA score	11 (1–20)	11 (3–21)	0.655	1/453
SAPS III Score	61 (25–104)	67 (15–99)	0.005	1/453
CPR before ECMO initiation	91 (25.6)	36 (36.7)	0.032	0/453
ECMO duration (days)	6 (1–20)	5 (1–22)	0.979	0/453
ICU length of stay (days)	17 (1–170)	18 (2–58)	0.275	0/453
Postcardiotomy				0/453
Postcardiotomy	243 (68.5)	54 (55.1)	0.016	
No cardiotomy	112 (31.5)	44 (44.9)		
Reason for ECMO support termination				0/453
Improvement	262 (73.8)	66 (67.3)	0.529	
Death	63 (17.7)	23 (23.5)		
Successful bridging	24 (6.8)	8 (8.2)		
Haemorrhage	6 (1.7)	1 (1.0)		
Complications				0/453
Haemorrhage	175 (49.3)	34 (34.7)	0.012	
Thrombosis	90 (25.4)	24 (24.5)	0.896	
Sepsis	62 (17.5)	21 (21.4)	0.379	
Mortality-related outcomes				0/453
Death on ECMO	62 (17.5)	24 (24.5)	0.145	
ICU-mortality	119 (33.5)	46 (46.9)	0.018	
In-hospital mortality	131 (36.9)	48 (49.0)	0.036	
1-year mortality	136 (38.3)	49 (50.0)	0.048	
Cause of death				0/453
Multiple organ failure	37 (28.5)	19 (42.2)	0.166	
Cardiac cause	62 (47.7)	13 (28.9)		
Respiratory failure	4 (3.1)	1 (2.2)		
Sepsis	15 (11.5)	5 (11.1)		
Brain death	12 (9.2)	7 (15.6)		

Data presented as mean ± standard deviation, median (minimum – maximum range) or number of patients (%). Abbreviations: SAPS III, simplified acute physiology score III; SOFA, sequential organ failure assessment score; ICU, intensive care unit; ECMO, extracorporeal membrane oxygenation; CPR, cardiopulmonary resuscitation.

Journal of Clinical Medicine

MDPI

Table S5. Selected demographic and clinical characteristics of patients according to the cause of cardiogenic shock (n = 453).

Patient characteristics	No cardiotomy		Postcardiotomy				Missing data (n/total)
	Acute heart failure (n = 126)	Right heart failure (n = 30)	Coronary artery bypass surgery (n = 66)	Heart valve surgery (n = 164)	Combined (n = 43)	Chronic heart failure (n = 24)	
Age (years)	56.6 ±13.3	56.8 ±13.9	67.7 ±9.0	63.3 ±15.1	62.9 ±11.1	49.1 ±15.3	0/453
Male sex	95 (75.4)	18 (60.0)	52 (78.8)	101 (61.6)	28 (65.1)	20 (83.3)	0/453
Body mass index (kg/m^2)	26.8 ±3.9	27.3 ±6.1	26.2 ±4.3	26.3 ±5.0	27.0 ±4.1	26.0 ±4.6	5/453
SOFA score	11 (1–21)	9.5 (5–18)	12 (4–19)	11 (3–20)	12 (3–20)	8 (2–17)	1/453
SAPS III Score	68 (37–104)	66 (40–97)	60 (31–101)	60 (28–98)	58 (31–89)	49 (15–84)	1/453
ECMO duration (days)	6 (1–17)	6 (1–17)	6 (1–22)	6 (1–20)	5 (1–16)	6 (2–17)	0/453
ICU length of stay	14 (1–121)	13 (2–92)	17 (1–59)	18 (1–170)	19 (2–55)	25 (2–90)	0/453
CPR before ECMO initiation	66 (52.4)	6 (20.0)	15 (22.7)	31 (18.9)	4 (9.3)	5 (20.8)	0/453
Weekend	41 (32.5)	3 (10.0)	18 (27.3)	18 (11.0)	9 (20.9)	9 (37.5)	0/453
Complications and mortality-related outcomes							0/453
Bleeding event	61 (48.4)	14 (46.7)	30 (45.5)	67 (40.9)	25 (58.1)	12 (50.0)	
Major bleeding event	16 (12.7)	7 (23.3)	18 (27.3)	54 (32.9)	16 (37.2)	8 (33.3)	
Thrombosis	35 (27.8)	9 (30.0)	13 (19.7)	40 (24.4)	11 (25.6)	6 (25.0)	
Sepsis	24 (19.0)	5 (16.7)	14 (21.2)	25 (15.3)	7 (16.3)	8 (33.3)	
Death on ECMO	32 (25.4)	9 (30.0)	12 (18.2)	23 (14.0)	7 (16.3)	3 (12.5)	
ICU-mortality	57 (45.2)	13 (43.3)	29 (43.9)	46 (28.0)	14 (32.6)	6 (25.0)	
3-months mortality	63 (50.0)	13 (43.3)	31 (47.0)	51 (31.1)	14 (32.6)	6 (25.0)	
One-year mortality	65 (51.6)	15 (50.0)	32 (48.5)	52 (31.7)	15 (34.9)	6 (25.0)	
Cause of death							0/453
Multiple organ failure	15 (25.9)	4 (28.6)	10 (32.3)	19 (36.5)	5 (37.5)	3 (50.0)	
Cardiac decompensation	28 (48.3)	6 (42.9)	13 (41.9)	22 (42.3)	5 (35.7)	1 (16.7)	
Respiratory failure	0 (0.0)	2 (14.3)	0 (0.0)	3 (5.8)	0 (0.0)	0 (0.0)	
Sepsis	5 (8.6)	0 (0.0)	6 (19.4)	6 (11.5)	2 (14.3)	1 (16.7)	
Brain death	10 (17.2)	2 (14.3)	2 (6.5)	2 (3.8)	2 (14.3)	1 (16.7)	
Other	6 (5.7)	1 (3.2)	0 (0.0)	10 (36.5)	10 (36.5)	10 (36.5)	

Data presented as mean ± standard deviation, median (minimum – maximum range) or number of patients (%). Abbreviations: SAPS III, simplified acute physiology score III; SOFA, sequential organ failure assessment score; ICU, intensive care unit; ECMO, extracorporeal membrane oxygenation; CPR, cardiopulmonary resuscitation.
